# Physical Activity Intensity and Learning Strategies in Students Aged 10 to 16: A Pilot Study

**DOI:** 10.3390/sports13030068

**Published:** 2025-02-25

**Authors:** Jose Luis Solas-Martínez, Alba Rusillo-Magdaleno, Alberto Ruiz-Ariza, Emilio J. Martínez-López

**Affiliations:** Musical, Plastic and Corporal Expression Didactics Department, Faculty of Humanities and Educational Sciences, University of Jaen, 23071 Jaén, Spain; jsolas@ujaen.es (J.L.S.-M.); arariza@ujaen.es (A.R.-A.); emilioml@ujaen.es (E.J.M.-L.)

**Keywords:** adolescents, children, cognition, heart rate, learning, physical exercise

## Abstract

This study analyzed the relationship between time spent on daily physical activity at different intensities (light, moderate, and vigorous) and learning strategies in adolescents. The sample included 147 Spanish adolescents (62% girls, 13.61 ± 1.95 years). Learning strategies were assessed using the *Motivated Strategies for Learning Questionnaire* (MSLQ), while physical activity intensity was recorded via the Xiaomi Mi Band 4 smartband, which measured heart rate (HR). HR was categorized into light (rest–50% max HR), moderate (50–70% max HR), and vigorous (70–85% max HR). Adolescents who engaged in moderate-intensity activities for 46–62 min daily showed the highest scores in elaboration, critical thinking, and metacognitive self-regulation, with girls also excelling in effort regulation (all *p* < 0.05). Among boys, 3–6 min of vigorous activity per day was linked to higher scores in elaboration, organization, critical thinking, peer learning, and rehearsal. Conversely, girls engaging in less than 3 min of vigorous activity obtained the highest scores in critical thinking and peer learning (all *p* < 0.05). It is recommended that students engage in 60 min of daily moderate-intensity physical activity. Additionally, short 5-min vigorous-intensity sessions should be encouraged in both boys and girls to enhance learning benefits and reduce social barriers to high-intensity exercise.

## 1. Introduction

**Figure 1:**
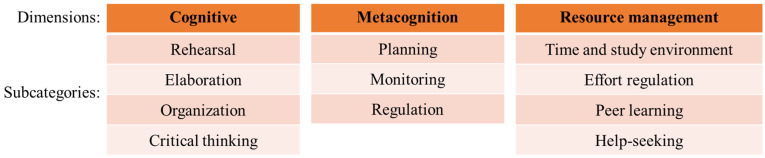
Dimensions and subcategories of learning strategies: cognitive, metacognitive, and resource management.

Achieving satisfactory academic performance is the primary goal of educational institutions [1]. Among the factors most strongly correlated with academic success, learning strategies play a crucial role in how young students navigate the educational process [2,3,4]. Learning strategies refer to the processes or sequences of processes that students intentionally employ to efficiently meet the demands of academic tasks [5]. These strategies involve tactical approaches that utilize cognitive tools, including behaviors, beliefs, and emotions, to integrate new information into existing knowledge and facilitate information retrieval [6,7]. Such methodological actions structure information processing, enabling key activities such as task planning, goal setting, progress monitoring, making adjustments, and evaluating both the process and outcomes [5]. Learning strategies are typically categorized into three main dimensions: cognitive, metacognitive, and resource management strategies [8,9] (Figure 1).

Cognitive strategies, as the first dimension, are associated with information processing and utilization and are employed during learning to enhance comprehension of a given subject [5,6]. These strategies can be classified into four main subcategories: rehearsal, elaboration, organization, and critical thinking [10]. Rehearsal involves reviewing or repeating previously studied information through recitation, reading, or practice. Elaboration facilitates the storage of new information in long-term memory by creating connections with prior knowledge. Organization aids in selecting relevant information and establishing relationships between concepts to be learned. Finally, critical thinking refers to the extent to which prior knowledge is applied to new situations to solve problems, make decisions, or conduct critical evaluations based on standards of excellence [10,11].

The second dimension, metacognition, refers to the ability to reflect on, understand, and regulate one’s own learning, encompassing both awareness and control of cognitive processes [8,9]. It includes key components such as planning, which involves setting objectives and analyzing tasks to activate or prepare relevant prior knowledge, thereby facilitating the organization and comprehension of new material [8]; monitoring, which refers to maintaining active attention during reading while continuously evaluating comprehension to ensure that new information is correctly assimilated and effectively connected to prior knowledge [9]; and regulation, which entails the continuous adjustment of cognitive activities, enabling progress monitoring, error identification, and correction throughout the learning process [12]. For example, while a cognitive strategy such as elaboration helps students create meaningful connections between concepts, a metacognitive strategy like monitoring allows them to assess whether those connections are aiding their understanding and adjust their study approach accordingly [5,9].

Finally, the third dimension, resource management strategies, are employed to control contextual factors that directly influence the learning process [5]. These strategies can be classified into four subcategories: time and study environment regulation, effort regulation, peer learning, and help-seeking from classmates or teachers [13]. Time management involves organizing and allocating study time based on planning and priorities [14]. Environmental management focuses on creating an appropriate, distraction-free space that enhances concentration and learning [15]. Effort regulation reflects the commitment to achieving academic goals despite challenges or distractions [16]. Peer learning involves engaging in discussions with classmates to support each other in understanding material and acquiring new knowledge [16]. Finally, help-seeking refers to reaching out to peers or teachers to resolve doubts, clarify concepts, and receive guidance when encountering difficulties during the learning process [17].

Some researchers argue that students must possess sufficient knowledge of these strategies, as well as the motivation to apply them effectively [5]. In the study by Trigueros & Navarro [18], it was observed that certain learning strategies are influenced by the satisfaction of psychological needs and students’ perceived autonomy in task execution. Additionally, academic self-efficacy and positive emotions have been identified as key determinants in the use of learning strategies [19]. In this context, regular physical activity emerges as a valuable tool for enhancing learning strategies and improving academic performance. Various studies have demonstrated its numerous benefits for executive functions, which are essential for planning, decision-making, and behavioral regulation [20,21]. Moreover, sustained engagement in physical activity over several weeks has been shown to enhance attention, memory, and readiness to learn [22]. It also contributes to emotional well-being by reducing anxiety, depression, and stress, as it strengthens social skills and self-concept [23]. Previous research has indicated that even a single session of physical activity can increase physiological arousal, thereby enhancing immediate attention and cognitive performance [20]. From a neurophysiological perspective, higher aerobic fitness is associated with better performance in tasks involving interference control, cognitive flexibility, and language and mathematical processing. Additionally, long-term physical activity programs stimulate neurotrophic factors that promote neural blood vessel formation and neurogenesis [20]. At a physiological level, physical activity has been shown to enhance brain function through multiple mechanisms, including increased cerebral blood flow and neurotrophic factor release, which support cognitive function and learning processes [24,25].

In this context, scientific literature highlights that the intensity at which physical activity is performed can significantly influence not only physical and psychological health but also various aspects of learning, such as memory, attention, and emotional regulation [26,27]. Light or low-intensity activities, such as walking, stretching, or practicing yoga, appear to contribute to stress reduction and emotional regulation [28]. These activities promote controlled nervous system activation and the release of neurotransmitters at lower levels, enhancing concentration and the ability to process and consolidate information [28]. Regarding moderate-intensity physical activity, evidence suggests improvements in selective attention, information encoding, and the reduction of mental fatigue [29]. Additionally, moderate-intensity exercise helps maintain a balance between physiological activation and cognitive demands, thereby promoting sustained concentration [30]. In contrast, vigorous-intensity physical activity has been linked to more pronounced neurobiological changes and stimulates the production of lactate, which serves as an important energy source for neurons and has been associated with both neuroprotective and cognitive-enhancing effects [25]. Furthermore, moderate- and vigorous-intensity exercise contribute to improved oxygenation and nutrient delivery to the brain, which supports synaptic plasticity and executive function [24]. These effects include increased release of neurotrophic factors that stimulate neural plasticity, facilitating working memory and executive function [31]. Consequently, planning, decision-making, and metacognitive regulation are potentially reinforced, contributing to enhanced academic performance [32].

On the other hand, previous studies have highlighted gender differences in the relationships between physical activity, cognitive processes, and academic performance, particularly due to varying levels of engagement during adolescence [33,34]. This developmental stage is especially relevant, as it marks a critical period for cognitive and emotional growth, characterized by the maturation of executive functions and the refinement of self-regulated learning processes [12]. Boys tend to participate more in sports and vigorous activities, as these practices are more socially accepted for them, whereas girls face sociocultural barriers that limit their participation [33,35]. Additionally, girls tend to invest less effort and involvement in physical or sports activities due to a lower self-perception of physical competence and more restrictive social encouragement [34]. As a result, boys often dominate spaces designated for physical activity, such as recess and team sports, while girls are frequently marginalized or receive less encouragement in mixed-gender settings, including Physical Education classes [34]. Similarly, significant gender differences have been observed in cognitive and academic domains. Girls generally excel in linguistic abilities and memory-related tasks, whereas boys outperform in spatial and mathematical skills [36]. While these disparities may have a biological component, they are strongly shaped by gender norms and expectations, which influence behavior and educational aspirations [36,37]. Girls are typically encouraged to develop qualities such as organization and attention to detail, which foster a more structured and methodical approach to learning [36]. In contrast, boys tend to adopt more competitive and independent patterns, displaying a more impulsive approach to academic tasks [36]. Moreover, girls often exhibit a greater inclination toward collaboration and mutual support in group contexts, whereas boys generally favor more competitive dynamics, which not only influence the intensity of their physical activity but also shape their approach to academic challenges [37].

Despite the aforementioned findings, the potential relationship between physical activity intensity levels and the use of learning strategies remains unclear. Various studies have reported mixed results regarding the influence of physical activity at different intensities on cognitive performance [38,39]. It appears that interventions incorporating higher-intensity physical activity, particularly when guided by qualified Physical Education professionals, produce greater positive effects on students’ academic performance [40]. However, no specific information is available regarding the role that each learning strategy may play in this relationship. Therefore, the objective of this study was to analyze the association between the time spent on daily physical activity at different intensity levels (light, moderate, and vigorous) and the use of learning strategies in students aged 10 to 16 years. It was hypothesized that regular engagement in moderate- or vigorous-intensity physical activity could be associated with higher levels of learning strategy use.

## 2. Materials and Methods

### 2.1. Participants

A total of 147 Spanish adolescents (62% girls and 38% boys) from five educational centers in Andalusia participated in this cross-sectional quantitative study. The students were between 10 and 16 years old (13.61 ± 1.95 years) and had a Body Mass Index (BMI) of 22.42 ± 3.50 kg/m^2^. Significant gender differences were observed in the use of the rehearsal (*p* = 0.024) and organization (*p* < 0.001) learning strategies. The sample was selected through convenience sampling from the participating schools. The anthropometric and sociodemographic characteristics of the participants are detailed in Table 1.

### 2.2. Measures

#### 2.2.1. Dependent Variables: Students’ Learning Strategies

Learning strategies were assessed using the Motivated Strategies for Learning Questionnaire (MSLQ) [13]. This self-report instrument consists of 81 items distributed across 15 subscales, evaluating both students’ motivational orientations toward course content and their use of different learning strategies. For this study, only the learning strategies section was used, which includes 31 items, along with an additional 19 items related to students’ resource management. These items are grouped into a total of nine subscales: (1) rehearsal, (2) elaboration, (3) organization, (4) critical thinking, (5) metacognitive self-regulation, (6) time and study environment regulation, (7) effort regulation, (8) peer learning, and (9) help-seeking from classmates or teachers. Responses were recorded using a seven-point Likert scale ranging from 1 (“Completely false for me”) to 7 (“Completely true for me”).

In the study by Ramírez et al. [41], where they validated an adaptation of the MSLQ questionnaire in university population, acceptable reliability levels were obtained (Cronbach’s α = 0.63 and 0.87, respectively). In the present study, after conducting a reliability analysis of the selected factors, the internal consistency of all factors was found to be acceptable (Cronbach’s α = 0.69–0.84), except for Factor 9 (Cronbach’s α = 0.47), which showed a value that was too low and was therefore excluded from the statistical analysis.

#### 2.2.2. Independent Variables: Daily Physical Activity Intensity

Daily physical activity intensity was calculated based on participants’ heart rate data recorded using the Xiaomi Mi Band 4 smartband. The study by de la Casa Pérez et al. [42] examined the validity and reliability of this device by comparing its data with video recordings in laboratory conditions and measurements from Sense Wear and Firstbeat monitors in free-living conditions. Their findings concluded that the Xiaomi Mi Band 4 provides acceptable validity and accuracy for measuring step count and daily heart rate in free-living conditions, both indoors and outdoors.

The smartband differentiates between daytime and sleep heart rate measurements. However, for this study, only daytime heart rate data were considered, while sleep time was excluded from the analysis. Additionally, the classification criteria established by the American Heart Association [43] were followed, defining light heart rate (HR) as the range between resting HR and 50% of maximum HR, moderate HR as 50–70% of maximum HR, and vigorous HR as 70–85% of maximum HR.

#### 2.2.3. Confounding Variables

Previous studies have concluded that age and BMI influence the level of systematic physical activity among adolescents [44]. Additionally, research has shown that mental health, intelligence quotient, and academic performance are associated with parental educational level, particularly that of mothers [45]. Therefore, in this study, age, BMI, and maternal education and employment status were considered as confounding variables.

Body weight and height were measured using an ASIMED^®^ Type B, Class III digital scale and a SECA^®^ 214 portable stadiometer (SECA Ltd., Hamburg, Germany). All measurements were taken with participants wearing light clothing and no footwear. Body Mass Index (BMI) was calculated as a derived variable using the formula: BMI = weight (kg)/height^2^ (m).

Sociodemographic data were collected using a structured questionnaire. This questionnaire included specific questions regarding participants’ age and school grade, as well as family-related aspects such as maternal education level and employment status. Additionally, the question “*How many computers are there in your home?*” was used as an indicator of the family’s economic status.

### 2.3. Procedure

The study was presented to the directors of the participating schools, and parental or legal guardian consent was obtained for minors. To ensure anonymity and confidentiality, each participant was assigned a coded identifier. Data collection began in February and concluded in May 2023, taking place during school hours designated specifically for the research. The data collection process was divided into two main phases. In the first phase, heart rate was measured over a full week using *Xiaomi Mi Band 4* smartwatches (Xiaomi Corporation, Beijing, China). Each participant received a smartwatch configured with their height, weight, and age and was instructed to wear it on their left wrist at all times for nine consecutive days. On the ninth day, the devices were collected, and heart rate data from a complete week were exported. Only participants who recorded valid data for seven full days (24 h per day) were included in the analysis. The devices were worn for nine days because the first and last days were not considered in the analysis, as participants received the smartwatch after starting their first day and returned it before completing their ninth day. Therefore, data from days 2 to 8 were used to ensure complete 24-h recordings. This process was repeated until data from all participants had been gathered. In the second phase, learning strategies were assessed, and sociodemographic data were collected using structured questionnaires.

### 2.4. Statistical Analysis

The results are presented as means and standard deviations, as well as percentages. Comparisons by sex for biometric characteristics and continuous sociodemographic variables were conducted using an independent samples *t*-test, while categorical sociodemographic variables were analyzed using the Chi-square test. The internal consistency of the MSLQ subscales was assessed using Cronbach’s alpha, calculated through inter-item correlations to ensure overall reliability. The recorded time (in minutes) for the three independent variables (light, moderate, and vigorous HR) was recoded into three groups based on the time spent in each heart rate interval, with categorization performed using the 33rd and 66th percentiles. The same method was applied to classify the data, resulting in the following groups for each heart rate category: for light HR, short time (<14 h 42 min/day), mid time (14 h 42 min–15 h 30 min/day), and long time (>15 h 30 min/day); for moderate HR, short time (<46 min/day), mid time (46–62 min/day), and long time (>62 min/day); and for vigorous HR, short time (<3 min/day), mid time (3–6 min/day), and long time (>6 min/day). To analyze differences between groups based on time spent in each heart rate interval (light, moderate, and vigorous HR) in relation to each factor of the MSLQ, a univariate ANCOVA was performed, with each MSLQ factor used as the dependent variable and the time spent in each heart rate interval and daily physical activity as fixed factors. Age, BMI, maternal education level, and maternal employment status were included as covariates. Each analysis was conducted separately and stratified by sex, given that previous research has reported sex differences in physical activity levels and academic performance [46,47]. A confidence level of 95% was applied for all analyses, which were conducted using the SPSS software package (version 25.0 for Windows, IBM Corp., Armonk, NY, USA).

## 3. Results

### 3.1. Association Between Physical Activity Intensity at Light HR (Resting HR—50% of Maximum HR) and Learning Strategies

**Figure 2:**
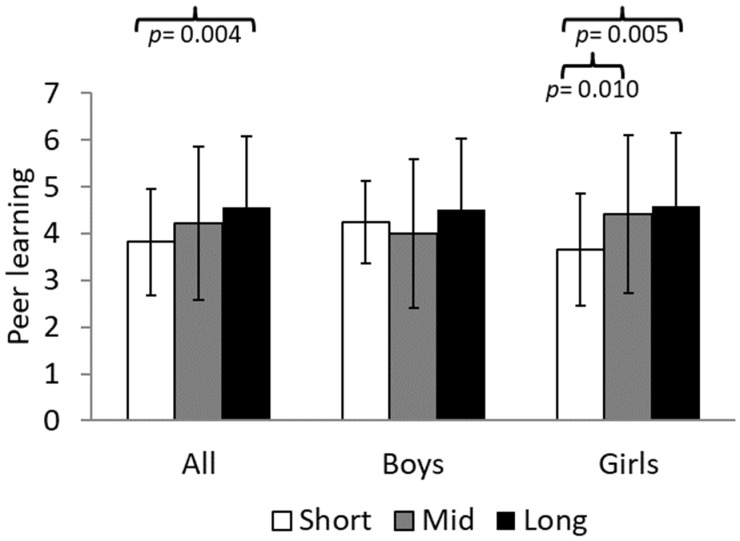
Comparison between the groups that spent short time (<14 h 42 min per day) vs. mid time (14 h 42 min–15 h 30 min per day) vs. long time (>15 h 30 min per day) in light HR with the peer learning factor of the MSLQ differentiated by sex.

The significant results from the ANCOVA analysis examining the relationship between daily physical activity at light HR and learning strategies are presented in Figure 2. For all participants, significant differences were found only in peer learning, F(2,139) = 4.341; Eta^2^ = 0.059; 1-β = 0.745 (*p* = 0.015). Participants who spent a long time at light HR obtained the highest scores compared to those in the short time category (Figure 2, 4.55 ± 1.53 vs. 3.82 ± 1.14 u.a., *p* = 0.004). When data were analyzed by sex, it was observed that girls who spent a long time at light HR achieved the highest peer learning scores, followed closely by those in the mid time category, and finally by those in the short time category, F(2,83) = 5.006; Eta^2^ = 0.108; 1-β = 0.801 (*p* = 0.009). A more detailed analysis revealed that girls in the long time category scored significantly higher than those in the short time category (Figure 2, 4.58 ± 1.52 vs. 3.66 ± 1.20 u.a., *p* = 0.005), while those in the mid time category also showed higher scores than those in the short time category (4.41 ± 1.69 vs. 3.66 ± 1.20 u.a., *p* = 0.010). Conversely, in the case of boys, no significant differences were found for this factor (all *p* > 0.05).

### 3.2. Association Between Physical Activity Intensity at Moderate HR (50–70% of Maximum HR) and Learning Strategies

**Figure 3:**
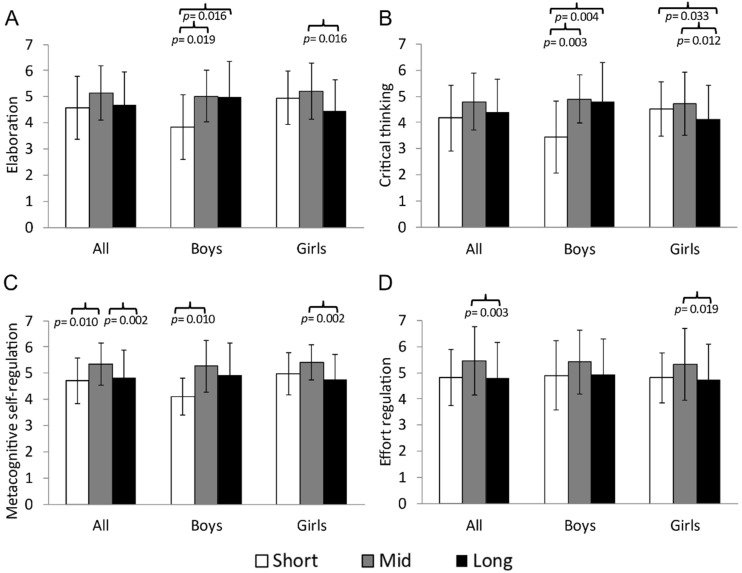
Comparison between the groups that were short time (<46 min/day) vs. mid time (46–62 min/day) vs. long time (>62 min/day) at moderate HR with the factors of (**A**) elaboration, (**B**) critical thinking, (**C**) metacognitive self-regulation and (**D**) effort regulation of the MSLQ differentiated by sex.

The significant results from the ANCOVA analysis examining the relationship between time spent at moderate HR and the factors of the MSLQ are presented in Figure 3. For all participants, those who spent mid time at moderate HR achieved significantly the highest scores in metacognitive self-regulation, F(2,138) = 5.801; Eta^2^ = 0.078; 1-β = 0.864 (*p* = 0.004), and effort regulation, F(2,138) = 4.632; Eta^2^ = 0.063; 1-β = 0.774 (*p* = 0.011). In pairwise comparisons, participants who spent mid time at moderate HR had significantly higher scores than those who spent long time at the same intensity in metacognitive self-regulation (Figure 3C, 5.35 ± 0.81 vs. 4.82 ± 1.06 u.a., *p* = 0.002) and effort regulation (Figure 3D, 5.46 ± 1.31 vs. 4.81 ± 1.33 u.a., *p* = 0.003). However, those who spent mid time at moderate HR only showed significantly higher scores than those who spent short time at this intensity in metacognitive self-regulation (Figure 3, 5.35 ± 0.81 vs. 4.70 ± 0.87 u.a., *p* = 0.010).

When analyzing the data by sex, boys who spent mid time at moderate HR obtained the highest scores in elaboration, F(2,48) = 3.674; Eta^2^ = 0.133; 1-β = 0.649, *p* = 0.033, critical thinking, F(2,48) = 5.731; Eta^2^ = 0.193; 1-β = 0.844, *p* = 0.006, and metacognitive self-regulation, F(2,48) = 3.636; Eta^2^ = 0.132; 1-β = 0.644, *p* = 0.034, followed by those in the long time category, who obtained similar values (*p* > 0.05). However, boys who spent mid time at moderate HR had significantly higher scores than those who spent short time at this intensity in elaboration (Figure 3A, 5.03 ± 0.99 vs. 3.83 ± 1.23 u.a., *p* = 0.019), critical thinking (Figure 3B, 4.90 ± 0.92 vs. 3.43 ± 1.37 u.a., *p* = 0.003), and metacognitive self-regulation (Figure 3C, 5.27 ± 0.99 vs. 4.13 ± 0.70 u.a., *p* = 0.010). Further pairwise analyses showed that boys who spent long time at moderate HR scored significantly higher than those in the short time category at this intensity in elaboration (Figure 3A, 5.00 ± 1.36 vs. 3.83 ± 1.23 u.a., *p* = 0.016), critical thinking (Figure 3B, 4.80 ± 1.48 vs. 3.43 ± 1.37 u.a., *p* = 0.004), and metacognitive self-regulation (Figure 3C, 4.94 ± 1.20 vs. 4.12 ± 0.70 u.a., *p* = 0.060).

On the other hand, girls who spent mid time at moderate HR obtained significantly the highest scores in critical thinking, F(2,83) = 5.377; Eta^2^ = 0.087; 1-β = 0.695, *p* = 0.023, and metacognitive self-regulation, F(2,83) = 5.082; Eta^2^ = 0.109; 1-β = 0.807, *p* = 0.008. In pairwise comparisons, girls in the mid time category at moderate HR had significantly higher scores than those in the long time category at this intensity in elaboration (Figure 3A, 5.21 ± 1.07 vs. 4.48 ± 1.17 u.a., *p* = 0.016), critical thinking (Figure 3B, 4.51 ± 1.17 vs. 4.29 ± 1.38 u.a., *p* = 0.012), metacognitive self-regulation (Figure 3C, 5.40 ± 0.67 vs. 4.75 ± 0.98 u.a., *p* = 0.002), and effort regulation (Figure 3D, 5.33 ± 1.38 vs. 4.73 ± 1.34 u.a., *p* = 0.019). Finally, girls who spent short time at moderate HR obtained significantly higher scores than those in the long time category at this intensity in critical thinking (Figure 3B, 4.52 ± 1.03 vs. 4.12 ± 1.30 u.a., *p* = 0.033).

### 3.3. Association Between Physical Activity Intensity at Vigorous HR (70–85% of Maximum HR) and Cognitive Learning Strategies

**Figure 4:**
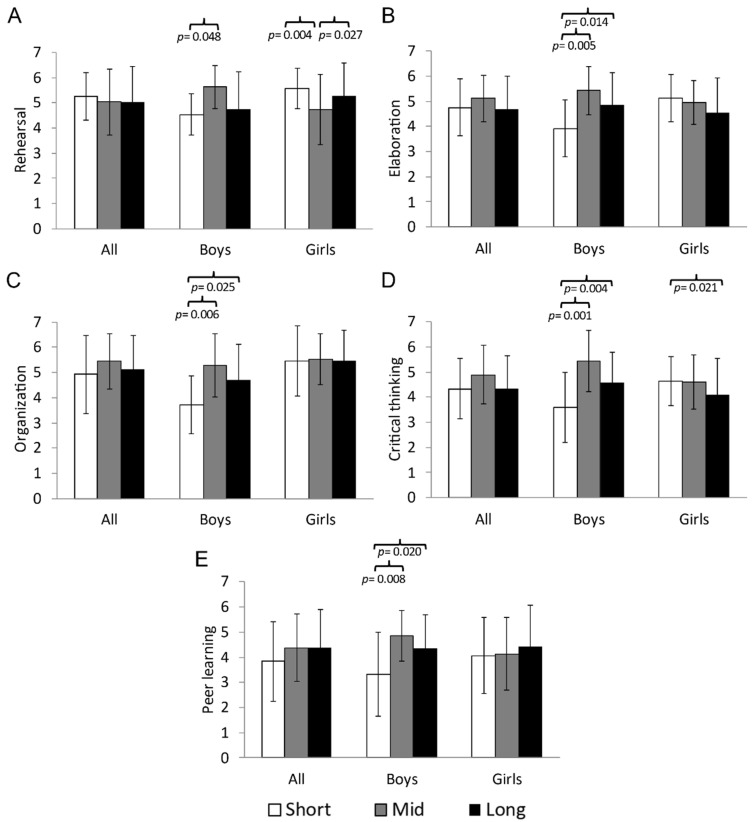
Comparison between the groups that spent short time (<3 min/day) vs. mid time (3–6 min/day) vs. long time (>6 min/day) at vigorous HR with the factors of (**A**) rehearsal, (**B**) elaboration, (**C**) organization, (**D**) critical thinking, and (**E**) peer learning of the MSLQ differentiated by gender.

The significant results from the ANCOVA analysis examining the relationship between time spent at vigorous HR and the factors of the MSLQ are presented in Figure 4. For all participants, no significant differences were observed in any of the learning strategies (*p* > 0.05). However, when analyzing the data by sex, boys who spent mid time at vigorous HR obtained the highest scores in elaboration, F(2,49) = 4.795; Eta^2^ = 0.164; 1-β = 0.771 (*p* = 0.013), organization, F(2,49) = 4.458; Eta^2^ = 0.154; 1-β = 0.739 (*p* = 0.017), critical thinking, F(2,49) = 7.164; Eta^2^ = 0.226; 1-β = 0.918 (*p* = 0.002), and peer learning, F(2,49) = 4.340; Eta^2^ = 0.150; 1-β = 0.727 (*p* = 0.018). The analysis also showed that boys who spent long time at vigorous HR obtained significantly higher scores compared to those in the short time category in rehearsal (Figure 4A, 5.63 ± 0.86 vs. 4.54 ± 0.83 u.a., *p* = 0.048), elaboration (Figure 4B, 5.43 ± 0.95 vs. 3.91 ± 1.13 u.a., *p* = 0.005), organization (Figure 4C, 5.29 ± 1.26 vs. 3.71 ± 1.14 u.a., *p* = 0.006), critical thinking (Figure 4D, 5.43 ± 1.21 vs. 3.60 ± 1.39 u.a., *p* = 0.001), and peer learning (Figure 4E, 4.86 ± 1.01 vs. 3.33 ± 1.67 u.a., *p* = 0.008). Furthermore, boys in the long time category at vigorous HR also achieved significantly higher scores than those in the short time category at this intensity in elaboration (Figure 4B, 3.91 ± 1.13 vs. 4.86 ± 1.28 u.a., *p* = 0.014), organization (Figure 4C, 3.71 ± 1.14 vs. 4.70 ± 1.40 u.a., *p* = 0.025), critical thinking (Figure 4D, 3.60 ± 1.39 vs. 4.59 ± 1.20 u.a., *p* = 0.004), and peer learning (Figure 4E, 3.33 ± 1.67 vs. 4.35 ± 1.35 u.a., *p* = 0.020).

On the other hand, girls who spent short time at vigorous HR obtained significantly the highest scores in rehearsal, F(2,83) = 4.660; Eta^2^ = 0.101; 1-β = 0.770 (*p* = 0.012), and critical thinking, F(2,83) = 3.200; Eta^2^ = 0.072; 1-β = 0.597 (*p* = 0.046). Pairwise analysis revealed that girls in the mid time category had significantly higher scores than those in the long time category at vigorous HR in rehearsal (Figure 4A, 4.73 ± 1.39 vs. 5.28 ± 1.31 u.a., *p* = 0.027). Additionally, it was found that girls who spent short time at vigorous HR achieved significantly higher scores than those in the long time category at this intensity in critical thinking (Figure 4D, 4.65 ± 0.97 vs. 4.11 ± 1.42 u.a., *p* = 0.021). Finally, girls in the short time category at vigorous HR obtained significantly higher scores than those in the mid time category at this intensity in rehearsal (Figure 4A, 5.57 ± 0.81 vs. 4.73 ± 1.39 u.a., *p* = 0.004).

## 4. Discussion

The main objective of this study was to evaluate the potential relationship between the time spent on daily physical activity at different intensities (light, moderate, and vigorous) and the use of learning strategies in adolescents. The variables that showed the most significant results were the time spent at moderate and vigorous HR. Regarding light HR, it was observed that girls who spent long time at this intensity obtained the highest scores in peer learning, followed closely by those in the mid time category, who achieved slightly lower values. On the other hand, both boys and girls who spent mid time at moderate HR achieved significantly better results in elaboration, critical thinking, and metacognitive self-regulation, with girls also excelling in effort regulation. As for vigorous HR, boys in the mid time category obtained significantly higher scores in rehearsal, elaboration, organization, critical thinking, and peer learning, whereas, in girls, those who spent short time at vigorous HR achieved significantly better scores in critical thinking and peer learning.

Both boys and girls who engaged in moderate HR activities for approximately 60 min per day (mid time) obtained the best results in learning strategies, significantly outperforming those who spent short or long time at this intensity. These findings align with previous studies that highlight the positive effects of moderate-to-vigorous physical activity on executive functions such as metacognitive regulation, sustained attention, and cognitive flexibility [20,21,48]. These benefits not only enable students to approach their academic tasks more strategically but also help them better organize and reflect on their learning processes [49]. Specifically, recent research has identified that regular engagement in moderate-intensity physical activity enhances specific skills related to metacognitive planning, such as prioritizing relevant information and anticipating learning difficulties [50]. Furthermore, similar to the findings of this study, Kim et al. [51] determined that the optimal weekly range of physical activity for improving both cognitive performance and mental health is between 2.5 and 7.5 h. This range maximizes the balance between adequate physiological activation and sustained cognitive capacity during learning; however, as indicated by the present study, insufficient or excessive time spent on physical activity reduces its positive effects on learning [52].

On the other hand, regarding the time spent at vigorous HR, our results reveal significant sex differences. Among boys, those who spent a mid time (between 3 and 6 min per day) engaging in high-intensity activities achieved the highest scores in learning strategies, followed by those who spent a prolonged time, whose values were similar. These findings align with studies highlighting how vigorous physical activity, even for short durations, is associated with improvements in attention regulation and memory consolidation due to its ability to enhance neurophysiological activation and cerebral blood flow [53]. Among girls, those who spent a short time at vigorous HR achieved the highest scores in rehearsal and critical thinking. These differences may be explained by the fact that women tend to experience a greater perception of fatigue and stress during vigorous activities, which may be attributed to factors such as increased sensitivity to muscle pain and a less efficient cardiovascular response under high-intensity loads [33]. As a result, less efficient regulation of mental and physical fatigue in such activities may interfere with key processes such as concentration and memory [54]. Additionally, sociocultural factors, such as lower familiarity with vigorous training and a less supportive environment for girls in high-intensity activities, create both psychological and physiological barriers that may limit their ability to apply learning strategies [55]. Consequently, girls do not experience the same benefits from physical activity as boys, despite the fact that the physiological changes resulting from this intensity are similar in both sexes [56]. To reduce these barriers and maximize the cognitive effects of exercise on their learning strategies, various studies suggest the need to eliminate social and psychological obstacles that limit their participation in high-intensity activities [33].

Sex differences in the results obtained at vigorous HR align with the findings of Asztalos et al. [35], who reported that the optimal intensity of physical activity for men is high, whereas for women it is moderate. It appears that boys are capable of maintaining more stable performance even under high-fatigue conditions due to greater activation in brain regions associated with motor control and response to physical exertion [54]. In contrast, girls tend to be more influenced by factors such as hormonal fluctuations and lower metabolic adaptation during vigorous activities, which increases their perception of effort [57]. Additionally, girls exhibit slower recovery after such activities, particularly in terms of muscle strength restoration and energy replenishment [58]. Another relevant factor is the pattern of cerebral blood flow redistribution in response to vigorous exercise. In boys, this redistribution favors areas associated with executive functions, optimizing cognitive processes, whereas in girls, the response tends to be more conservative, prioritizing energy homeostasis mechanisms over cognitive performance [59].

### 4.1. Recommendations

Based on the findings of this study, it is recommended that both boys and girls engage in approximately 60 min of daily moderate-intensity physical activity, such as brisk walking or recreational sports, as this is associated with significant improvements in learning strategies related to elaboration, critical thinking, metacognitive self-regulation, and effort regulation. Additionally, for boys, complementing these sessions with short bouts of vigorous physical activity, such as running or high-intensity training, may further enhance the effects of physical activity on rehearsal, elaboration, organization, critical thinking, and peer learning. For families, it is essential to foster an environment that encourages regular physical activity, ensuring that adolescents have sufficient time and opportunities to engage in moderate-to-vigorous exercise without exceeding a duration that leads to fatigue. Schools should consider implementing structured physical activity programs within the curriculum, including both moderate and vigorous activities, to maximize cognitive and academic benefits. Educators, in turn, could incorporate active breaks and moderate-to-vigorous physical exercises into the school schedule, leveraging their potential to optimize learning and improve academic performance. Furthermore, it is crucial to raise awareness and provide support for girls in engaging in high-intensity physical activities, overcoming potential sociocultural barriers that may limit their participation. Policymakers should promote initiatives that integrate physical activity into educational strategies, ensuring that schools receive the necessary resources to implement evidence-based interventions that enhance learning through movement.

### 4.2. Limitations and Strenghts

The main limitation of this study is the relatively small sample size for a cross-sectional study, as it is not representative of the general population and was selected through convenience sampling. Although the data cannot be generalized, we believe they provide valuable insights as a pilot study. Additionally, the classification of the three groups based on the 33rd and 66th percentiles (short, mid, and long time) for the independent variables according to the time spent in each heart rate interval (light, moderate, and vigorous) may not accurately reflect the distribution of the studied population. This classification was applied uniformly across the entire sample using the same cutoff points for boys and girls, even though these values might differ depending on sex. Also, a limitation of the Xiaomi Mi Band 4 smart wristband is its tendency to slightly overlap Light HR data between wakefulness and sleep, leading to an estimated overestimation of ~5%. However, this issue does not affect Moderate and Vigorous HR data. Lastly, since this is a cross-sectional study, it is not possible to establish causal relationships between learning strategies and physical activity. Despite these limitations, the study presents several strengths, including the use of instruments with high reliability and validated internal consistency, as well as the rigorous adherence to the research protocol across all participating centers. Moreover, several influential covariates related to education and physical activity in children and adolescents were considered, such as age, BMI, and maternal education and employment status.

## 5. Conclusions

It is concluded that engaging in moderate-intensity physical activity for approximately 60 min per day is positively associated with learning strategies related to elaboration, critical thinking, and metacognitive self-regulation in both boys and girls, and with effort regulation exclusively in girls. Additionally, in boys, spending as little as 5 min per day on vigorous-intensity physical activity may be sufficient to enhance learning factors such as rehearsal, elaboration, organization, critical thinking, and peer learning. It is recommended to promote educational and family programs that encourage regular physical activity, with at least one hour per day at moderate-to-vigorous intensity. Furthermore, brief sessions of vigorous physical activity (5 min per day) have shown an impact on the use of learning strategies and could be implemented during class breaks or recess. Finally, raising awareness about these associations between physical activity and learning could serve as an additional stimulus toward academic success.

## Figures and Tables

**Table 1:** Biometric characteristics, sociodemographic data, and average results of minutes at each heart rate interval (light, moderate, and vigorous) and MSLQ factors segmented by sex.

	All (n = 147)		Boys (n = 56)		Girls (n = 91)		
Variables	Mean	SD	Mean	SD	Mean	SD	*p*
**Age (years)**	13.61	1.95	13.60	2.03	13.61	1.88	0.956
**Weight (kg)**	52.30	12.94	54.84	15.56	50.09	9.66	**0.005**
**Height (m)**	1.59	0.12	1.61	0.14	1.57	0.082	**0.014**
**BMI (kg/m^2^)**	20.42	3.5	20.76	3.87	20.12	3.13	0.156
**No. of computers**	2.05	1.38	1.98	1.28	2.10	1.47	0.488
**Mother’s level of education (%)**							0.163
No education	7.6%		2.8%		4.8%		
Primary	19.3%		9.6%		9.6%		
Secondary	16.5%		5.6%		10.8%		
Vocational training	14.1%		5.6%		8.4%		
University	24.1%		11.2%		12.9%		
Don’t know	18.5%		11.2%		7.2%		
**Mother’s employment status (%)**							0.979
Not working	22.4%		10.2%		12.2%		
Part-time	28.9%		13%		15.9%		
Full-time	45.9%		22%		24%		
Retired	2.8%		1.2%		1.6%		
**Average PA**	3.85	1.82	4.09	1.87	3.66	1.76	0.100
**Min. per day at light HR**	1001.60	93.57	1008.29	90.75	996.24	95.85	0.369
**Min. per day at moderate HR**	66.97	26.96	66.36	25.69	67.45	28.03	0.776
**Min. per day at vigorous HR**	7.3	7.89	8.44	9.69	6.39	5.98	0.084
**MSLQ**							
Rehearsal	5.11	1.24	4.88	1.22	5.28	1.24	**0.024**
Elaboration	4.80	1.16	4.71	1.19	4.86	1.32	0.363
Organization	5.14	1.33	4.67	1.32	5.50	1.22	**<0.001**
Critical thinking	4.57	1.23	4.59	1.26	4.56	1.22	0.870
Metacognitive self-regulation	4.98	0.91	4.87	0.97	5.06	0.86	0.163
Time and Study environment	5.03	1.04	4.91	0.90	5.12	1.13	0.142
Effort regulation	5.02	1.22	5.03	1.24	5.02	1.21	0.960
Peer learning	4.27	1.48	4.29	1.38	4.26	1.56	0.890
Help seeking	4.66	0.96	4.71	0.94	4.63	0.97	0.577

Notes. Data are presented as means for continuous variables and frequencies (%) for categorical variables. BMI = body mass index; SD = standard deviation; PA = physical activity; HR = heart rate; MSLQ = Motivated Strategies for Learning Questionnaire.

## Data Availability

Data are unavailable due to privacy restrictions.
